# Advances on Bioactive Metabolites with Potential for the Biocontrol of Plant Pathogenic Bacteria †

**DOI:** 10.3390/pathogens13111000

**Published:** 2024-11-15

**Authors:** Pierluigi Reveglia, Gaetano Corso, Antonio Evidente

**Affiliations:** 1 Department of Clinical and Experimental Medicine, University of Foggia, Viale Pinto 1, 71121 Foggia, Italy; pierluigi.reveglia@unifg.it (P.R.); gaetano.corso@unifg.it (G.C.); 2 Institute of Biomoleular Chemistry National Research Council, Via Campi Flegrei 34, 80078 Pozzuoli, Italy

**Keywords:** plant pathogens, bacteria, biocontrol, natural substances

## Abstract

The increase in the world population, which will be almost 10 billion by 2050, will require considerable efforts to significantly increase food production. Despite the considerable progress made in agriculture, this need is becoming an emergency due to desertification, environmental pollution and climate changes. Biotic stresses, such as pathogenic bacteria and fungi, primarily contribute to significant losses in agricultural productivity and compromise food safety. These harmful agents are predominantly managed using large quantities of synthetic pesticides. However, this widespread use has led to substantial environmental pollution, increased pest resistance and toxic residues in agricultural produce, which subsequently enter the food supply, posing severe health risks to humans and animals. These challenges have significantly driven the advancement of integrated pest management strategies to reduce or eliminate synthetic pesticides. A practical and viable alternative lies in biopesticides—methods developed from natural products that are safe for human and animal health. This approach aligns with the strong demand from consumers and public authorities for safer pest control solutions. This review was focused on the isolation, chemical and biological characterization of natural products for the biocontrol of phytopathogenic bacteria and, in some cases, fungi with potential eco-friendly applications.

## 1. Introduction

Phytopathogenic bacteria and fungi produce different phytotoxins that are involved in heavy diseases that seriously damage agrarian, forest and ornamental plants [1,2,3]. Several studies have been carried out on the role of bioactive microbial metabolites in the pathogenic process and on their potential application in agriculture [4,5,6,7,8]. These studies have allowed the scientists to identify substances which are not only essential for agriculture but also have potential applications in other fields. Microbial bioactive metabolites, including phytohormones, phytoalexins, antibiotics, fungicides, herbicides and elicitors, belong to several classes of natural compounds of low molecular weight (amino acids, aromatic compounds, anthraquinone, naphthoquinone, terpenes, macrolides, furanones, cytokinins, auxins, etc.) as well as high molecular weight (proteins, glycoproteins and more recently polysaccharides) [1,2,9,10,11,12]. One of the main applications of bioactive microbial metabolites is to control weed and parasitic plant diffusion, which are a severe problem for crops and pastures, as well as for forest heritage and ornamental plants [11,13,14]. Today, the control of pests is reached via the extensive use of chemicals; pesticides, which, when applied several times, cause an increase in pest resistance in hosts; high environmental pollution and serious problems to human and animal health [15,16]. Despite the significant rise in pesticide usage, crop losses have remained relatively high over the past 40 years. This approach has yet to enable farmers to adapt production systems or effectively increase crop productivity, often resulting in greater vulnerability to pest damage [17]. In contrast, employing biological control agents—such as specific insects and bacterial or fungal pathogens—alongside natural phytotoxins offers an environmentally compatible, highly targeted and sustainable long-term solution. Some reviews concerning these aspects have been published [11,14,18,19].

Metabolites from different natural sources, with potential activity against bacteria inducing severe diseases to agrarian and forest plants, are considered an important group of new potential bactericides. Thus, some reviews have treated natural bactericides but only partially dealing with this argument. Among them, there is a review dealing with the metabolites produced by genus *Xylaria* Hill (e.g., Schrank, 1789, Xylariaceae), which includes various endophytic fungi species. Certain fungi produce a variety of natural compounds with potential applications such as herbicides, fungicides and insecticides; others demonstrate antibacterial, antimalarial, antifungal and α-glucosidase inhibitory activities, showing promise for use in both agriculture and medicine [20]. Another study focused on bacteriocins, antimicrobial substances produced by many bacteria, including lactic acid bacteria (LAB), which are effective against various saprophytic and pathogenic microorganisms. This review emphasized the role of immobilized bacteriocins from LAB, highlighting their significance in natural food preservation and shelf-life extension, their use in health care for creating probiotic foods and beverages, their potential as antibiotic alternatives in clinical settings and their application as biocontrol agents against plant pathogens in agriculture [21]. Another review treated bioactive metabolites, and, in particular, pantocin A produced by *Pantoea* species, which was evaluated as a biocontrol agent for fire blight disease of apple and pear [22]. Surprisingly, seaweeds also are a source of bioactive compounds, already used in different industries and with antibiotic activity against several phytopathogens agents. Among these compounds, some are identified for their eliciting ability to trigger a priming defense mechanism [23]. Successively, a review discussed cyanobacteria as organisms with significant potential in agriculture as biopesticides. In fact, they produce different biological active compounds that have a demonstrated efficacy as antibacterial, antiviral, antifungal, insecticidal, herbicidal and more [24].

Considering the serious diseases induced by phytopathogenic bacteria and their consequent heavy economic losses, in the present review, the natural compounds with antibacterial activity obtained from different sources (bacteria, fungi and plants) are reported. In particular, the treatment was focused on their chemical and biological properties and on their potential application as bacteriocides in agriculture. The results discussed in the three different sections were obtained from Sci-Finder research and chronologically reported. The results of the SARs (structure–activity relationships) studies, as well as the mode of action of some specialized metabolites and their efficacy against severe and specific plant diseases, have also been discussed.

## 2. Bactericides from Bacteria

*Pseudomonas savastanoi* pv. *savastanoi* (*Ps. savastanoi*), is a very harmful phytopathogen bacterium and is a common resident in the olive phyllosphere. This bacterium invades the host tissues by penetrating through wounds of various natures, inducing the formation of nodes. The virulence factors produced by the bacterium, including indole-3-acetic acid (IAA) [25], various cytokinins [26] and hrp genes [27], play a fundamental role in the process of node differentiation. The *Pseudomonas syringae* pv. *ciccaronei* strain NCPPB2355 produced a bacteriocin capable of inhibiting the growth of the *Ps. savastanoi* strain. The bacteriocin was characterized as a protein that was revealed in the three SDS (sodium dodecyl sulfate)-PAGE bands with molecular weights of 76, 63 and 45 kDa (kDalton), respectively, and that was resistant to non-polar organic solvents and active under neutral conditions [28]. Subsequently, this bacteriocin was tested at different levels of purification and concentrations in culture and in plants, showing a significant inhibition of the multiplication of *Ps. savastanoi*. The bacteriocin treatments inhibited the formation of olive nodes in the plant induced by different *Ps. savastanoi* strains. The same bacteriocin was also effective in controlling the multiplication of epiphytic pathogen populations, as the bacterial populations recovered after 30 days were at least 350 and 20 times lower than the control populations on twigs and leaves, respectively [29].

**Figure 1:**
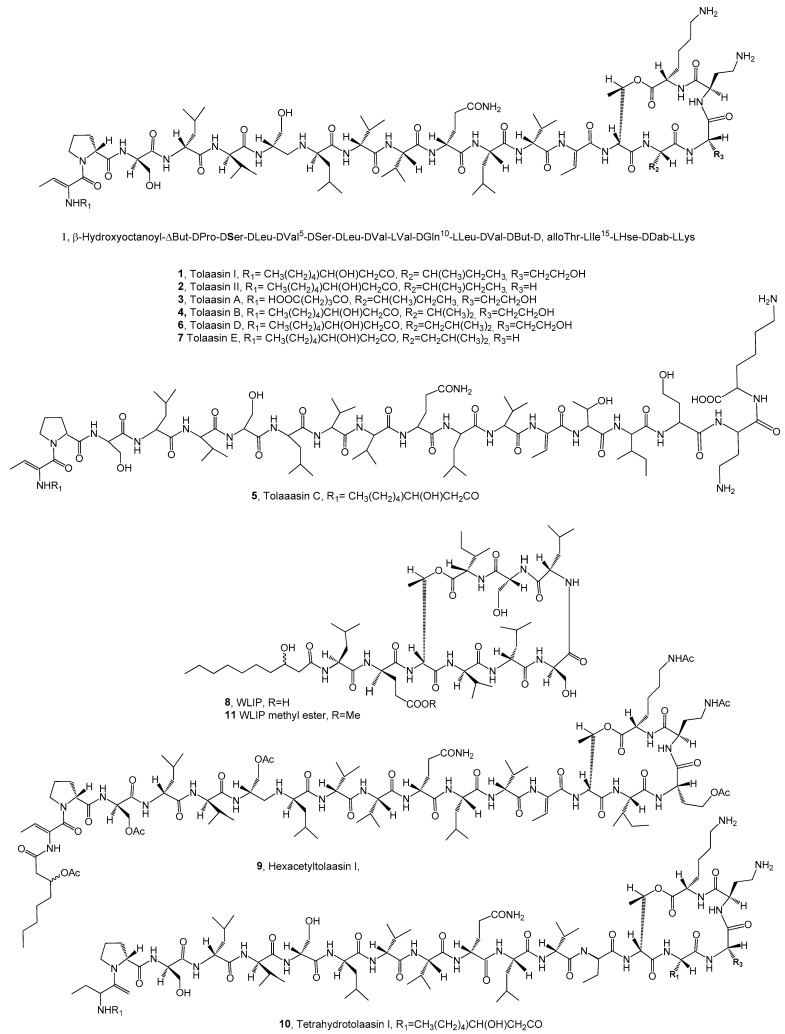

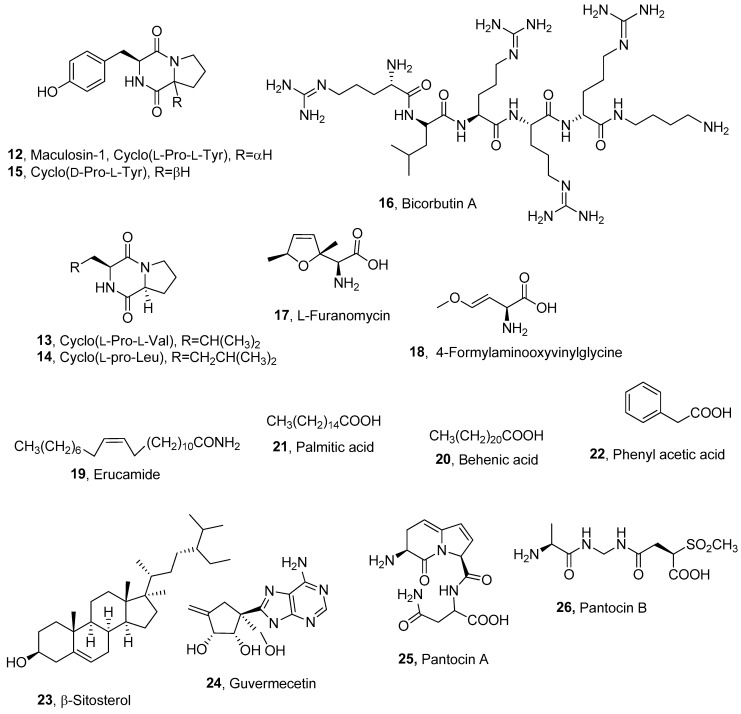
Bacterial metabolites with potential for the biocontrol of plant pathogenic bacteria.

Tolaasin I, tolaasin II and five additional minor analogs—designated as tolaasins A, B, C, D and E (compounds **1**–**7**, Figure 1, Table 1)—are part of the lipodepsipeptide (LPD) group [30] and are produced by *Pseudomonas tolaasii*, the pathogen responsible for brown blotch disease in *Agaricus bisporus* as well as the yellowing of *Pleurotus ostreatus*. The antimicrobial effects of tolaasins (**3**–**7**) were evaluated alongside tolaasin I and II (**1** and **2**) against several organisms: the yeast *Rhodotorula pilimanae*, the fungus *Rhizoctonia solani*, Gram-positive bacteria like *Bacillus megaterium* and *Rhodococcus fascians* and Gram-negative bacteria like *Escherichia coli* and *Erwinia carotovora* subsp. *carotovora*. The results indicated that *B. megaterium* and *R. fascians* were the most sensitive microorganisms as, except for tolaasin C, all the LPDs tested inhibited the growth of these bacteria, but the differences among their specific activities were observed. Tolaasin D (**6**) was the most potent compound, followed by tolaasin I and II (**1** and **2**), with minimal inhibitory quantities of 0.16, 0.32 and 0.64 µg, respectively. In contrast, tolaasins A, B and E (**3**, **4** and **7**) exhibited lower activity, with minimal inhibitory quantities of 1.28 and 2.56 µg, respectively. The fungus *R.nia solani* showed a similar sensitivity to these compounds. None of the LPDs inhibited the growth of Gram-negative bacteria, such as *E. coli* and *E. carotovora* subsp. *carotovora* at the concentrations tested. However, tolaasins I, II and D (**1**, **2** and **6**) effectively inhibited the growth of *R. pilimanae* [31].

Considering the results of SAR studies, the lactone ring and the *N*-terminus acyl moiety appeared to be important to impart the antimicrobial activity of tolaasins compared to tolaasin I (**1**) and tolaasin A (**3**), which contain pentanedioic acid instead of β-hydroxy octanoic acid, demonstrating reduced activity, while tolaasin C (**5**), the linear form of **1** that lacks the lactone ring, shows no activity. Additionally, the substituent at position C-15 plays a significant role in inhibitory activity. Specifically, replacing isoleucine at position 15 with valine or leucine, as seen in tolaasins B and D (**4** and **6**), led to a decrease or increase in antimicrobial activity relative to compound 1. Furthermore, leucine at position 15, as in tolaasin E (**7**), reduced activity compared to compound **2**. Although this effect appears contradictory when comparing tolaasin D (**6**), compound **2** differed from **1** due to the substitution of homoserine with glycine at position 16 [31]. The LPD, defined as the L-Inducing Principle (WLIP, **8**, Figure 1, Table 1), produced by *Pseudomonas reactans* NCPPB1311, is known as an inductor in the “white line” assay for the specific identification of *P. tolaasii* [32]. WLIP (**8**) should be regarded as an actual toxin, as it inhibits the growth of fungi—including cultivated mushrooms such as *Agaricus bisporus*, *Lentinus edodes* and *Pleurotus* species—as well as Chromista and Gram-positive bacteria. Additionally, compound **8** inhibited the growth of *B. megaterium* ITM100 with a minimal inhibitory quantity (MIQ) of 0.32 μg and that of fungi, Chromista and Gram-positive bacteria at M.I.Q. values higher than those of tolaasin I (**1**). LPD **8** did not inhibit the growth of most of the tested Gram-negative bacteria, with the only exception of the strain *E. carotovora* subsp. *carotovora*. When tested against blocks of *Agaricus bisporus* and compared to LPD 1, WLIP demonstrated lower activity in altering the mushroom’s pseudo-tissues. Both WLIP and tolaasin I were shown to cause red blood cell lysis through colloid–osmotic shock, mediated by the formation of transmembrane pores; however, WLIP exhibited greater hemolytic activity than tolaasin I. The antifungal properties of WLIP, along with the observation that a virulent morphological variant of *P. reactans* lacks WLIP production, suggest that WLIP may play a key role in the interactions between *P. reactans* and cultivated mushrooms [33].

The antimicrobial activities of five lipodepsipeptides (**1**, **2**, **6** and **7**); of the WLIP (**8**) of the two tolaasin I (**1**) derivatives, hexaacetyl and the tetrahydro-tolaasin I (**9** and **10**, Figure 1, Table 1) and of the methyl ester of the WLIP (**11**, Figure 1) were tested against several bacteria and fungi pathogenic of agrarian plants. In the same experiment, four 2,5-diketopiperazines (**12**, **13**, **14** and **15**, Figure 1, Table 1) were tested. Diketopiperazine (DKP), represents the smallest known class of cyclic peptides [34]. Among 2,5-diketopiperazines, the most known is maculosin-1 (cyclo(L-Pro-L-Tyr)) (**12**), which is a host specific phytotoxin produced by *Alternaria alternata*, a pathogen of knapweed [35]. DKP **12** was also recently isolated from *Lysobacter capsici* AZ78 and showed antifungal activity against *Phytophthora infestans* and *Plasmopara viticola*, both causal agents of important crop diseases [36]. LPDs and DKPs were assayed towards bacteria belonging to the *Pseudomonas* genus and the pathogens of important agrarian plants.

These bacteria include the following: *Burkholderia caryophylli* (syn. *Pseudomonas caryophylli*), which is the causal agent for bacterial wilt of carnation [37]; *P. syringae* pv. *panici*, which is a worldwide diffused pathogen of crops [38,39]; *Pseudomonas syringae* pv. *tabaci*, which induces brown spots on tobacco [40]; *P. syringae* pv. *siringae*, which is the most polyphagous bacterium in the *P. syringae* complex due to its wide host range, first affecting woody and herbaceous host plants [41] and *Pseudomonas syringae* pv. *japonica*, which causes the black node disease of barley (*Hordeum vulgare* L.) and wheat (*Triticum aestivum* L.) [42]. *B. subtilis*, *B. megaterium* and *E. coli*, which are laboratory strains, were also used. The test results showed that among the tolaasins and their two derivatives, the LPDs **1**, **2** and **6** and the tetrahydrotolaasin I (**10**) inhibited all the bacteria (MIC (minimum inhibition concentration) range of 0.1–0.9 μg/mL), while tolaasin E and hexacetyltolaasin I (**7** and **16**) showed a MIC in the range of 3–6 and 0.7–1 μg/mL, respectively. *E. coli* growth was not inhibited. The highest antimicrobial activity was exhibited by tolaasin D (**6**) (MIC range of 0.1−0.2 μg/mL) and the lesser toxicity was shown by tolaasin E (**7**) and the derivatives **9** and **10** (MIC range of 0.7–1.0 and 0.2–3.0 μg/mL). The amino acid residue at the C-16 position of the macrolactone ring seemed not to be important for the activity as LPDs **1** and **2** showed strong bactericidal activity. This SAR relation, as well as the others described above were confirmed. Furthermore, the acetylation of the hydroxyl groups of macrocyclic lactones and the hydrogenation of two residues of 2-butenylbutiric acids present in the derivatives **9** and **10**, significantly induced a decrease in the activity. The lack of toxicity of the WLIP (**8**) and its methyl ester (**11**) against all the phytopathogenic bacteria was probably due to strong structural differences with tolaasins. However, LPD **8** exhibited activity against the two laboratory Gram-positive strains *B. subtilis* and *B. megaterium*, except in compound **12** on *E. coli*, the results of the bioassays of the four DKPs (**12**–**15**) showed that almost all the dicyclopeptides had bactericidal activity against all the bacteria used. The 2,5-diketopiperazine compound **13** was not toxic. Among the active diketopiperazines (DKPs) **12**, **14** and **15**, the highest antimicrobial activity was observed in cyclo(L-Pro-L-Tyr), **12**, with a minimum inhibitory concentration (MIC) range of 15–20 μg/mL, while the other two compounds (**14** and **15**) exhibited lower activity, with MIC ranges of 500–800 μg/mL. The configuration of the amino acids, whether D or L, played a crucial role in determining activity. This was evident from the inactivity of compound **13** and the reduced activity of DKP **15**, which differed from **12** only by the opposite D stereochemistry of the proline residue, resulting in a 50–60 times decrease in activity compared to **12**. The noteworthy reduction in activity by 40–50 times showed by compound **14**, in respect to that of **12**, which differs for the substitution of L-Tyr with L-Leu, demonstrated that the nature of the amino acids, which constitute dicyclopeptides, also affected the activity [43].

Entomopathogenic bacteria (EPB) produce antibiotics that are effective against the fire blight bacterium *Erwinia amylovora*, including strains resistant to streptomycin, and demonstrate similar efficacy in phytotron experiments as kasugamycin or streptomycin. Among these EPB strains, *Xenorhabdus budapestensis* and *Xenorhabdus szentirmaii* produced antibiotics that inhibited colony formation and mycelial growth of *Phytophthora nicotianae*. Bicornutins A-C were isolated from *X. budapestensis* (**16**, Figure 1, Table 1) and were identified as new hexapeptides. While bicornutins B and C share similarities with bicornutin A, their exact structures have yet to be reported. Though in unknown ratios, ten preparations containing all three bicornutin compounds exhibited antibacterial activity against *B. subtilis*, with inhibition zones ranging from 13 to 24 mm. The same bicorbutin complex was also tested against *E. amylovora* at four concentrations and the bacterium appeared to be very susceptible to each of all the concentrations assayed [44].

L-Furanomycin [(2*S*,2′*R*,5′*S*)-2-amino-2-(5′methyl-2′,5′-dihydrofuran-2′-yl)]acetic acid (**17**, Figure 1, Table 1), a non-proteinogenic amino acid, was produced by *Pseudomonas fluorescens* SW25, a strain originally isolated from wheat rhizosphere [45]. Genetic studies demonstrate that the *P. fluorescences* SW25 is the closest strain to WH6, which produces another non-proteinogenic amino acid identified as 4-formylaminooxyvinylglycine (L-2-amino-4-formylaminooxy-*trans*-3-butenoic acid, FVG, **18**, Figure 1, Table 1). FVG (**18**) showed selective herbicidal and antimicrobial activities and irreversibly arrested the germination of a large number of graminaceous species, including a number of invasive grassy weeds [46]. Furthermore, compound **18** exhibited selective antimicrobial activity against some bacteria including *E. amylovora*, the causal agent of the disease of orchard crops known as fire blight [47]. The *P. fluorescence* strain SW25 produced, together with compound **17** and other non-proteinogenic amino acids such as FVG (**18**), while rhizobitoxine (4-(2-amino-3-hydroxypropoxy)vinylglycine, methoxyvinylglycine (MVG), L-2-amino-4-methoxy-*trans*-3-butenoic acid and 3-methylarginine were produced by *Pseudomonas. andropogonis* [48], *Pseudomonas aeruginosa* (ATCC-7700) [49] and *Pseudomonas syringae* pv. *syringae* [50], respectively. L-Furanomycin (**17**) inhibited the growth of several microorganisms as *Shigella paradysenteriae*, *Salmonella paratyphi A* and *B- subtilis* [51]. Other bacteria are susceptible to furanomycin, including several plant pathogens as *Dickeya dadantii*, *P. syringae* and *E. amylovora*, as well as the nonpathogenic strain of *B. megaterium* [45].

Erucamide, behenic, palmitic, phenylacetic acids and β-sitosterol (**19**–**23**, Figure 1, Table 1), were purified from the organic extract of *B. megaterium* and their activity was tested against *Agrobacterium tumefaciens* T-37, *E. carotovora* EC-1 and *Ralstonia solanacearum* RS-2. Palmitic acid (**21**) had no antibacterial activity (>500 µg/mL), while erucamide (**19**) showed moderate antibacterial activity (MIC 500 µg/mL). Behenic acid (**20**) was active with MICs of 250 µg/mL against T-37 and RS-2 strains while β-sitosterol (**23**) showed significant activity against RS-2. (MIC of 15.6 µg/mL). Phenylacetic acid (**22**) exhibited activity towards all the three strains T-37 and against EC-1 and RS-2 (with a MIC of 62.5, 125, 15.6 µg/mL, respectively) and showed potential for their biocontrol [52].

Guvermectin (GV, **24**, Figure 1, Table 1), is a *N*^9^–glucoside cytokinin derivative obtained from the purification of *Streptomyces sanjiangensis* NEAU6 extract (Liu et al., 2022 [53]. To evaluate the antibacterial activity of GV and its mechanism targeting GMPs (Guanosine 5′-monophosphate synthetase), an enzyme essential for bacterial guanine synthesis, various biochemical and genetic methods were employed, including enzyme activity assays, site-directed mutagenesis, bio-layer interferometry and molecular docking assays. The target bacteria included *R. solanacearum*, which affects many host plants, *Pseudomonas syringae* pv. *actinidiae*, the pathogen responsible for kiwifruit canker and *Xanthomonas oryzae* pv. *oryzae*, which causes significant yield reductions in rice (10–50%). The results indicated that GV effectively inhibits GMPs, disrupting bacterial guanine synthesis, thereby shedding light on the antibacterial mechanism of GV and its potential as a biocontrol agent in agriculture [54].

Pantocins A and B (**25** and **26**, Figure 1, Table 1), two peptides, were isolated from an endophytic strain, *Pantoea* PC-2B, derived from *Convolvulus arvensis* L., a prevalent weed in potato fields. The antagonistic activity of pantocins A and B was tested against *Pectobacterium carotovorum* subsp. *carotovorum* (Pcc), the pathogen responsible for potato tuber decay, resulting in an approximately 58.8% reduction in tuber decay in vivo. When the *Pantoea* strain was used for pre-treatment, it led to a 56.7% reduction in disease incidence and a 52% reduction in curative challenges during semi-practical storage trials. These findings suggest that the tested *Pantoea* strain may be a promising candidate for protecting potato tubers from soft rot disease caused by Pcc [55].

**Table 1:** Bacterial metabolites with potential for the biocontrol of plant pathogenic bacteria.

Compounds	Source	Bacterium Target	References
Bacteriocin	*Pseudomonas syringae* pv. *ciccaronei*	*Pseudomonas savastanoi* pv. *savastanoi*	[28,29]
Tolaasin I (1), Table 1	*Pseudomonas tolaasii*	*Bacillus megaterium* and *Rodococcus fascians*	[31]
		*Burkholderia caryophylli*, *P. syringae* pv. *panici*, *Pseudomonas syringae* pv. *tabaci*, *P. syringae* pv. *siringae* and *Pseudomonas syringae* pv. *japonica*, *B. subtilis*, *Bacillus megaterium*	[43]
Tolaasin II (2)	″	*Bacillus megaterium* and *Rodococcus fascians*	[31]
		*Burkholderia caryophylli*, *P. syringae* pv. *panici*, *Pseudomonas syringae* pv. *tabaci*, *P. syringae* pv. *siringae* and *Pseudomonas syringae* pv. *japonica*, *B. subtilis*, *Bacillus megaterium*	[43]
Tolaasins A (3)	″	*Bacillus megaterium* and *Rodococcus fascians*	[31]
Tolaasins B (4)	″	″	″
Tolaasins C (5)	″	″	″
Tolaasins D (6)	″	*Bacillus megaterium* and *Rodococcus fascians*	[31]
		*Burkholderia caryophylli*, *P. syringae* pv. *panici*, *Pseudomonas syringae* pv. *tabaci*, *P. syringae* pv. *siringae* and *Pseudomonas syringae* pv. *japonica*, *B. subtilis*, *Bacillus megaterium*	[43]
Tolaasins E (7)	″	*Bacillus megaterium* and *Rodococcus fascian*	[31]
		*Burkholderia caryophylli*, *P. syringae* pv. *panici*, *Pseudomonas syringae* pv. *tabaci*, *P. syringae* pv. *siringae* and *Pseudomonas syringae* pv. *japonica*	[43]
WLIP (8)	*Pseudomonas reactans*	*B. megaterium*, *Erwinia carotovora subsp. carotovora*	[33]
		*B. subtilis* and *B. megaterium*	[43]
Hexacetyltolaasin I (9)		*Burkholderia caryophylli*, *P. syringae* pv. *panici*, *Pseudomonas syringae* pv. *tabaci*, *P. syringae* pv. *siringae* and *Pseudomonas syringae* pv. *japonica*,	[43]
Tetrahydrotolaasin I (10)		*Burkholderia caryophylli*, *P. syringae* pv. *panici*, *Pseudomonas syringae* pv. *tabaci*, *P. syringae* pv. *siringae* and *Pseudomonas syringae* pv. *japonica*, *B. subtilis*, *Bacillus megateriums*	[43]
WLIP methyl ester (11)		*B. subtilis* and *B. megaterium*	″
Maculosin-1, Cyclo(L-Pro-L-Tyr) (12)	*Lysobacter capsici*	*Burkholderia caryophylli*, *P. syringae* pv. *panici*, *Pseudomonas syringae* pv. *tabaci*, *P. syringae* pv. *siringae* and *Pseudomonas syringae* pv. *japonica*, *B. subtilis*, *Bacillus megaterium*	″
Cyclo(L-Pro-L-Val) (13)	″	Not toxic	″
Cyclo(L-pro-Leu) (14)	″	*Burkholderia caryophylli*, *P. syringae* pv. *panici*, *Pseudomonas syringae* pv. *tabaci*, *P. syringae* pv. *siringae* and *Pseudomonas syringae* pv. *japonica*, *B. subtilis*, *Bacillus megaterium*, *E.coli*	″
Cyclo(D-Pro-L-Tyr) (15)	″	″	″
Bicornutin A (16)	*Xenorhabdus budapestensis* and *X. szentirmaii*	*Erwinia amylovora*	[44]
L-Furanomycin (17)	*Pseudomonas fluorescens* SW25	*Dickeya dadantii*, *P. syringae*, *E. amylovora* and *b. subtilis*	[45]
4-Formylaminooxyvinyl glycine (18)	*Pseudomonas fluorescens* WH6	*Erwinia amylovora*	[46,47]
Erucamide (19)	*Bacillus megaterium*	*Agrobacterium tumefaciens*, *Erwinia carotovora* and *Ralstonia solanacearum*	[52]
Behenic acid (20)	″	″	″
Palmitic acid (21)	″	Not toxic	″
Phenylacetic acid (22)	″	″	″
β-Sitosterol (23)	″	″	″
Guvermectin (24)		*Ralstonia solanacearum*, *Pseudomonas syringae pv. actinidiae Xanthomonas oryzae pv. oryzae*	[54]
Pantocin A (25)	*Pantoea* sp.	*Pectobacterium carotovorum* subsp. *carotovorum*	[55]
Pantocin B (26)	″	″	″

″ means the same content.

## 3. Bacteriocides from Fungi

**Figure 2:**
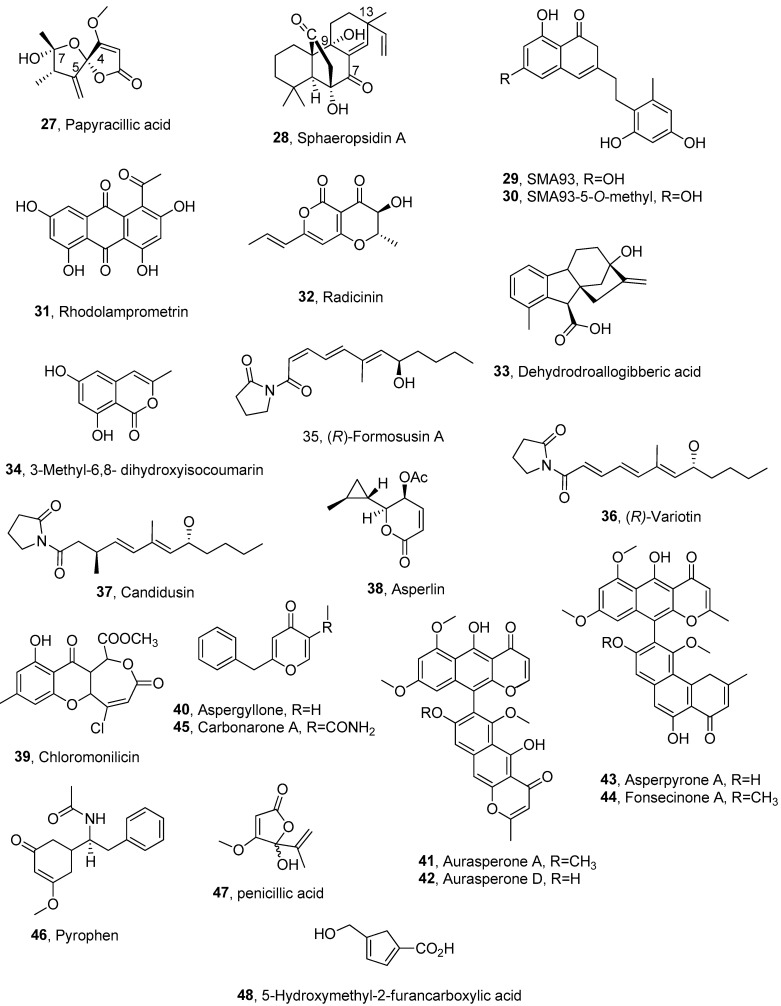
Fungal metabolites with potential for the biocontrol of plant pathogenic bacteria.

Papyracillic acid (PA, **27**, Figure 2, Table 2), a spiran complex toxin, was isolated as the main phytotoxin from a strain of *Ascochyta agropyrina* var. *nana*, which was proposed as bioherbicide to the control from *Elytrigia repens*. *E. repens* (quack grass) is a noxious perennial weed widespread through the cold regions of the northern and southern hemispheres.

PA (**27**) showed toxicity towards the Gram-positive and Gram-negative bacteria such as *B. subtilis* and *Xanthomonas campestris*, respectively, when tested at a range of concentrations from 1.5 to 100 μg/disk. Compound **27** was also toxic against the fungus *Candida tropicalis* at the concentration of 6.25 μg/disk [56]. Previously, papyracillic exhibited strong antimicrobial, nematicidal and cytotoxic activity against *Bacillus brevis*, *B. subtilis*, *Microcossus luteus* (Gram-positive) and *Enterobacter dissolvens* (Gram-negative) and against the fungi *Nematospora coryli*, *Mucor miehie*, *Penicillium notatum* and *Paecilomyces varioti* [57]. PA (**27**) also exhibited nematicidal activity against *Caenorhabditis elegans* and *Meloidogyne incognita* [58]. When tested at a range of concentrations of significant phytotoxic activity, a different sensitivity was observed. Some key derivatives of PA were prepared and used in a SAR study. PA (**27**) was converted to its methyl ester and methyl acetal by well-known reactions with diazomethane and acid-catalyzed methanol, respectively, while its acetylation afforded some acetyl derivatives, which resulted in an inseparable epimeric mixture of the two monoacetyl derivatives in a ratio of ca. 1:1. The other two acetylated compounds were a different monoacetyl and one of its diastereomers. In all the acetyl derivatives, a cyclopentenone ring was preserved. By catalytic hydrogenation, PA gave the expected dihydroderivative, with the saturation of the exocyclic methylene group. All the PA and its derivatives were tested using a leaf disk–puncture assay at 1 mg/mL concentration. The toxin was phytotoxic to both the host plant and several non-host plants, while its derivatives showed significantly reduced activity compared to the toxin (**27**). A structure–activity relationship (SAR) study revealed that the butenolide ring is a crucial component for phytotoxicity and that the exocyclic methylene group at C-5 also plays a role in inducing toxicity. In contrast, the tetrasubstituted tetrahydrofuran was found to be non-essential. The reduced activity of the PA acetal further suggests that the hemiacetalic hydroxy group at C-7 contributes to the toxin’s toxicity. As previously mentioned, the PA was also effective against *X. campestris*, *B. subtilis* and *C. tropicalis*, while all the derivatives in the same tests exhibited significantly lower toxicity than compound **27** [56].

Sphaeropsidin A (**28**, Figure 2, Table 2) is a tetracyclic pimarane diterpene produced as the main phytotoxin by *Diplodia cupressi* (syn. of *Sphaeropsis sapinea* f. sp. *cupressi*), which is the causal agent of the severe canker disease of Italian cypress (*Cupressus sempervirens* L.) in the Mediterranean basin. Other phytopathogen species of *Diplodia* and some endophytic fungal strain such as *Tubercularia* sp. and *Smardaea* sp. synthesize compound **28**. The latter toxin showed different and interesting biological activities, such as phytotoxic, antifungal and antibiotic activity with potential applications in agriculture as biopesticides (natural herbicides, fungicides and bacteriocides) and antiviral and anticancer activity, with potential in medicine to combat malaria, yellow fever and dengue. All the aspects of compound **28** including biosynthesis, isolation, characterization, biological activities, natural analogs, hemisynthetic derivatives and the results of some SAR studies have been recently and extensively reviewed [59].

More recently, some lipophilic derivatives of compound **28** were synthesized by modifying the C15 and C16–alkene moiety. Several of these derivatives induced significant endoplasmic reticulum (ER) swelling, associated with proteasomal solid inhibition and cell death—a mechanism not observed with the natural product itself. Analysis from the National Cancer Institute’s screening of sixty cell lines showed no correlations between the most potent derivative and other compounds in the database, except at high concentrations (LC50, Lethal Concentration 50%). This study developed a new set of sphaeropsidin derivatives that could be explored as potential anticancer agents, mainly due to their continued efficacy against multidrug-resistant models [60]. The authors Alexander Kornienko., Veronique Mathieu, Willem A. L. van Otterlo, Antonio Evidente, Aude Ingels A.I. and Sachin B. Wagh are the inventors of the patent application PCT/US23/35648 (date of application 21 October 2023). As regards a potential and significant application of diterpene **28** in agriculture, the toxin, sphaeropsidins B and C, two of its natural analogs, 14 of their derivatives, which were obtained by chemical modifications of the toxins were assayed for their antibacterial activity towards *Xanthomonas oryzae* pv. *oryzae*, *Pseudomonas fuscovaginae* and *Burkholderia glumae*, which are the causal agents of severe bacterial rice diseases. The proposed antibacterial activity of diterpene **28** and its natural analogs and derivatives is based on their structural similarity to oryzalexins A-D [61,62,63] and momilactones A and B [64], which are phytoalexins produced by *Oryza sativa * L. in response to attacks by *Pyricularia oryzae*. These compounds have potential applications in the control of plant diseases. Toxin **28** demonstrated specific and potent activity against *Xanthomonas oryzae* pv. *oryzae*, while no toxicity was observed against the other two pathogens. Structure–activity relationship (SAR) studies conducted with the cited derivatives of compound **28** indicated that the key structural features essential for antibacterial activity include the presence of the C-7 carbonyl group and hemiketal lactone functionalities. Additionally, the C-13 vinyl group, the double bond in ring C, the tertiary C-9 hydroxy group and the pimarane arrangement of the tricyclic carbon skeleton were also found to contribute significantly to the antibacterial properties [65].

SMA93, its 5-*O*-methyl, rhodolamprometrin, radicinin, dehydroallogibberic acid and 3-methyl-6,8-dihydroxyisocoumarin (**29**–**34**, Figure 2, Table 2) were isolated from *Fusarium proliferatum* ZS07, a fungus obtained from long-horned grasshoppers (*Tettigonia chinensis*). Compounds **30** and **32** inhibited the radicle growth of *A. retroflexus* L. seeds at a concentration of 100 μg/mL, with the inhibition rates of 83.0 and 65.2%, respectively. Compounds **29**–**31** exhibited antibacterial activity against *B. subtilis* (MIC values of 3.13–12.50 μg/mL) but had no effect towards *E. coli* and *Salmonella typhimurium* [66].

Sphearopsidin A (**28**) was also produced, together with (*R*)-formosusin A, (*R*)-variotin, candidusin and asperlin (**35**–**38**, Figure 2, Table 2) by *Aspergillus candidus* SFC20200425-M11, which has the potential to reduce the development of fungal plant diseases such as tomato late blight and wheat leaf rust. All the compounds were isolated, except compound **38**, showed antifungal activity against plant pathogenic fungi such as *Alternaria brassicicola*, *Botrytis cinerea*, *Colletotrichum coccodes*, *Fusarium oxysporum*, *Magnaporthe oryzae* and *Phytophthora infestans* (MIC ranging 1–250 μM/mL), but only asperlin (**38**) showed antibacterial activity against *Clavibacter michiganensis* and *E. amylovora* with MIC values of 125 and 250 μg/mL, respectively [67].

Chloromonilicin (**39**, Figure 2, Table 2) was isolated for the first time, along with the known phytotoxic polycyclic ethanones alternethanoxins A-E, from *Alternaria sonchi*, a mycoherbicide proposed for controlling the noxious weed *Sonchus arvensis*. Chloromonilicin (**39**) exhibited a broad spectrum of antimicrobial activity against bacteria, yeasts and plant pathogenic fungi. When tested against *B. subtilis*, *E. coli* and *P. fluorescens*, compound **39** showed a minimum inhibitory concentration (MIC) of less than 0.5 μg per disk. It was less effective against *Paenibacillus polymyxa* and *C. tropicalis*, with an MIC of around 1 μg per disk. Additionally, compound **39** inhibited the germination of conidia from four widely distributed plant pathogenic fungi—*Alternaria tenuissima*, *Bipolaris sorokiniana*, *Colletotrichum gloeosporioides* and *Fusarium culmorum*—with an MIC of less than 1 μg/mL. Notably, compound **39** did not exhibit phytotoxicity against couch grass’s perennial sow thistle or leaf segments. However, at a minimal concentration of 1 μg/mL, it inhibited the movement of *Paramecium caudatum* within 1 h of treatment. The ciliates were killed at higher concentrations (10 and 100 μg/mL) after 20 and 10 min of incubation, respectively [68].

Aspergyllone A (**40**, Figure 2, Table 2), a new 6-benzyl-γ-pyrone, was isolated together with the aurasperones A and D, asperpyrone A, fonsecinone A, carbonarone A and pyrophen (**41**–**46**, Figure 2, Table 2) from the culture filtrates of an endolichenic fungus *Aspergillus niger* Tiegh [69]. The fungus was collected from lichen thallus *Parmotrema ravum* (Krog and Swinscow) Serus, in India. All the compounds were assayed for their antimicrobial activity and aspergyllone (**40**) showed strong selective antifungal activity against *Candida parapsilosis* (IC_50_ of 52 mg/mL) and carbonarone A (**45**) exhibited activity against *Candida albicans* and *Candida krusei* (IC_50_ (inhibitory concentration, 50%) 103 mg/mL and 31 mg/mL, respectively). Compound **45** also showed significant activity against a plant pathogenic bacterium, *Dickeya solani* (IC_50_ 88 mg/mL), which is the causal agent of blackleg and slow wilt symptoms of potato plants in a number of European countries and Israel [70]. Aurasperone A (**41**) displayed antibacterial activity against *Pseudomonas aeruginosa* and *S. aureus* (IC_50_ of 160 mg/mL and 135 mg/mL, respectively) and anti-candidal activity only towards *C. krusei* (IC_50_ of 373 mg/mL). Fonsecinone A (**44**) inhibited only *S. aureus* and *E. coli* (IC_50_ of 120 mg/mL and 47 mg/mL, respectively) and the plant pathogen, *Pseudomonas syringae* pv. *maculicola* McCulloch with IC_50_ of 154 mg/mL. *P. s.* pv. *maculicola caused* leaf spot and blight diseases of crucifer crops worldwide [71]. Asperpyrone A (**43**) exhibited antibacterial activity against *E. coli*, with an IC50 value of 112 mg/mL. Pyrophen (**46**) demonstrated promising antimicrobial properties, inhibiting 50% of the tested organisms, including humans, fish and foodborne pathogens. It exhibited antifungal activity against various *Candida* species, except *C. krusei*. Furthermore, significant antibacterial activity was observed against *Micrococcus luteus* (IC50 63 mg/mL), *Aeromonas hydrophila* (IC50 78 mg/mL) and *Listeria innocua* (IC50 86 mg/mL). In contrast, Aurasperone D (**42**) showed no antimicrobial activity [69]. Penicillic acid (**47**, Figure 2, Table 2) was isolated from *Penicillium* sp. CRM 1540 obtained from Antarctic marine sediment as a potential bioinsecticide against *Xanthomonas citri* subsp. *citri*, which is the causal agent of citrus canker. When tested in vitro assays against *X.campestris* and *X. citri* subsp. *citri*, penicillic acid (**47**) showed MIC for 90% growth inhibition of the bacterial cells of 49.39 and 25.0 µg/mL, respectively. In greenhouse experiments, penicillic acid (25 µg/mL) suppressed citrus canker development by 75.31% [72].

5-Hydroxymethyl-2-furancarboxylic acid (**48**, Figure 2, Table 2), was isolated from *Aspergillus niger xj* together with ergosterol, β-sitosterol, 5-pentadecylresorcinol and succinimide [73]. All the compounds were tested against three plant pathogen bacteria, namely, *E. carotovora*, whose effect on plant were cited above [74]; *Agrobacterium tumefaciens*, which can infect 643 species of dicotyledonous plants and a few gymnosperm plants of 331 genera and 93 families [75]; *Ralstonia solanacearum*, which is the causal agent of potato brown rot, the bacterial wilt of tomato, tobacco, eggplant and some ornamental plants, as well as of the Moko disease in bananas [76]. Compound **48** exhibited the most potent antibacterial activity against the tested bacteria, with *R. solanacearum* being the most sensitive, showing the lowest MIC of 15.56 µg/mL. These results suggest that the mechanism of action of compound **48** against *R. solanacearum* may involve interference with bacterial protein synthesis and intracellular metabolism. This hypothesis is supported by observations from scanning electron microscopy, cell membrane permeability tests and SDS-PAGE analysis [73].

**Table 2:** Fungal metabolites with potential for the biocontrol of plant pathogenic bacteria.

Compounds	Source	Bacterium Target	References
Papyracillic acid (27)	*Ascochyta agropyrina* var. *nana*	*Bacillus subtilis*, *Xanthomonas campestris*, *Bacillus brevis*, *Microcossus luteus Enterobacter dissolvens*	[56]
Sphaeropsidin A (28)	*D. cupressi*	*Xanthomonas oryzae* pv. *oryzae*	[65]
SMA93 (29)	*Fusarium proliferatum*	*B. subtilis*	[66]
5-*O*-Methylated of SMA93 (30)	“	“	“
Rhodolamprometrin (31)	“	“	“
Radicinin (32)	“	Not toxic	“
Dehydrodroallogibberic acid (33),	“	Not toxic	“
3-Methyl-6,8- dihydroxyisocoumarin (34)	“	Not toxic	“
(*R*)-Formosusin A (35)	*Aspergillus candidus*	“	[67]
(*R*)-Variotin (36)	“	“	“
Candidusin (37),	“	“	“
Asperlin (38)	“	*Clavibacter michiganensis E*. *amylovora*	“
Chloromonilicin (39)	*Alternaria sonchi*	*B. subtilis*, *E. coli*, *P. fluorescens* and *Paenibacillus polymyxa*	[68]
Aspergillone (40)	*Aspergillus niger*	Not toxic	[69]
Aurasperone A (41)	“	*Pseudomonas aeruginosa* and *S. aureus*	“
Aurasperone D (42)	“	Not toxic	“
Asperpyrone A (43),	“	*E. coli*	“
Fonsecinone A (44)	“	*S. aureus*, *E. coli and Pseudomonas syringae* pv. *maculicola*	“
Carbonarone A (45)	“	*Dickeya solani*	“
Pyrophen (46)	“	*Micrococcus luteus*, *Aeromonas hydrophila* and *Listeria innocua*	“
Penicillic acid (47)	*Penicillium* sp.	*Xanthomonas citri* subsp. *citri*, *Xanthomonas campestris*	[72]
5-Hydroxymethyl-2-furancarboxylic acid (48)	*Aspergillus niger xj*	*Erwinia carotovora*, *Agrobacterium tumefaciens*, *Ralstonia solanacearum*	[73]

“ means the same content.

## 4. Bacteriocides from Plants

**Figure 3:**
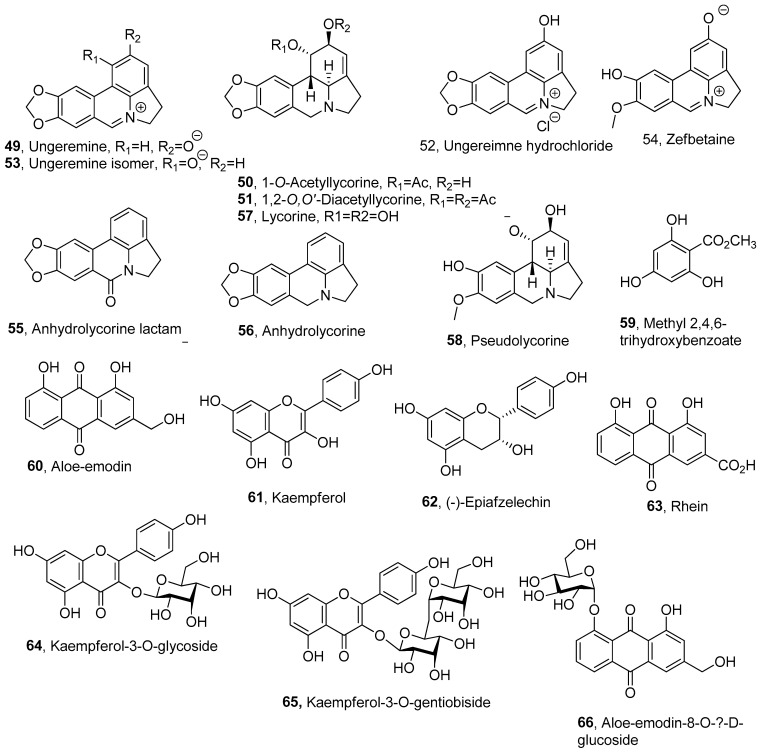
Plant metabolites with potential for the biocontrol of plant pathogenic bacteria.

The pond-raised channel catfish (*Ictalurus punctatus*) industry, has great economic importance in United States, particularly in the southeastern region. Thus, environmentally derived pre-harvest off-flavors, due to cyanobacterium *Planktothrix perornata* (Skuja) [77] and the bacterial disease columnaris, and the enteric septicemia of catfish (ESC) caused by *Flavobacterium columnare* and *Edwardsiella ictaluri*, respectively, resulted in significant economic losses [78]. The classic control methods have low efficacy and selectivity [79]. Ungeremine (**49**, Figure 3, Table 3), an alkaloid isolated from a variety of Amaryllidaceae plant species, including *Ungernia minor*, *Crinum americanum*, *Crinum asiaticum*, *Zephyranthes flava* and *Pancratium maritimum* [80], showed toxicity against *E. ictaluri* (IC_50_ and the MIC values were 58.0 and 3.0 mg/L, respectively). Ungeremine was also among the most toxic compounds towards *F. columnare*, as well as 1-*O*-acetyllycorine and 1,2-*O*,*O*’-diacetyllycorine (**50** and **51**, Figure 3, Table 3) [80], which are hemisynthetic derivatives of lycorine (**57**, Figure 3, Table 3), which is the main Amaryllidaceae alkaloid [81]. A SAR study was carried out using several derivatives of ungeremine and lycorine, such as ungeremine hydrochloride, ungeremine isomer, zefbetaine (**52**–**54**, Figure 3), anhydrolycorine lactame, anhydrolycorine and pseudolycorine (**55**, **56**, **58**, Figure 3), respectively, and testing their toxicity against *F. columnare*. The results revealed that the C ring’s aromatization and the B ring’s oxidation at C-7 to an azomethine group are critical structural features for antibacterial activity. Additionally, the position of the oxygenation on the C ring and the presence of the 1,3-dioxole ring attached to the A ring of the pyrrolo[de]phenanthridine skeleton also play significant roles in enhancing activity. Based on 24 h, 50% inhibition concentration (IC50) results, ungeremine hydrochloride (**52**) showed toxicity comparable to compound **49**, while alkaloid **55** demonstrated the lowest activity. The water solubility of analog **52** may enhance its potential as an effective feed additive, making it more practical than ungeemine [82].

The bioactivity of the metabolites of oregano (*Origanum vulgare*) essential oil, grown in the arid Andes region are not extensively studied. Thus, a study performed by GC-MS (Gas Chromatography–Mass Spectrometry) showed the presence in the essential oil of oregano, collected in the Atacama Desert, of the well-known thymol (15.9%) as the main metabolite, *Z*-sabinene hydrate (13.4%), γ-terpinene (10.6%), *p*-cymene (8.6%), linalyl acetate (7.2%), sabinene (6.5%) and carvacrol methyl ether (5.6%). This essential oil showed antibacterial activity towards *S. aureus* and *Salmonella enterica* and the phytopathogenic bacteria *Erwinia rhapontici* and *X. campestris*. Furthermore, oregano oil exhibited antibacterial activity against bacteria associated with food poisoning [83].

Methyl 2,4,6-trihydroxybenzoate, aloe-emodin, kaempferol, (-)-epiafzelechin, rhein, kaempferol-3-O-glycoside, kaempferol-3-O-gentiobiside and aloe-emodin-8-O-β-D-glucoside (**59**–**66**, Figure 3, Table 3) were isolated from the leaf extracts of *Cassia alata* L., which demonstrated significant efficacy against plant diseases caused by fungi such as *Magnaporthe oryzae*, *Phytophthora infestans*, *Colletotrichum coccodes* and *Puccinia recondita* in vivo. Among the isolated metabolites, compounds **61**–**66** exhibited in vitro antifungal activity against *M. oryzae* and *Phytophthora* species, with rhein (**63**) notably inhibiting the mycelial growth of *Phytophthora* species and effectively suppressing tomato late blight. Furthermore, compound **63** showed the potent in vitro inhibition of *Acidovorax avenae* subsp. *cattlvae* growth, with an IC50 of 2.5 μg/mL [84].

The *Tithonia diversifolia* leaves were macerated in water and the corresponding aqueous fraction and essential oil were shown to contain phenols, tannins, favonoids, alkaloids, terpenoids, sugars, saponins, hydrocarbonated and oxygenated monoterpenes, terpenoids and sesquiterpenes. α-Terpineol, eucalyptol, camphor and α-pinene (were the main compounds (20.3%, 14.6%, 14.3% and 13.5%, respectively). The aqueous extract inhibited *Bipolaris oryzae* and *Fusarium moniliforme* (IC_50_ 50 mg/mL), while the essential oil exhibited toxicity towards the phytopathogenic bacteria *X. oryzae* pv. *oryzae* and *Pseudomonas fuscovaginae* (MIC 125 μg/mL), and against the two cited fungi [85].

**Table 3:** Plant metabolites with potential for the biocontrol of plant pathogenic bacteria.

Compounds	Source	Bacterium Target	References
Ungeremine (49)	*Pancratium maritimum*	*Edwardsiella ictaluri*, *Flavobacterium columnare*	[80]
1-*O*-Acetyllycorine (50)	“	“	“
1,2-*O*,*O*’-Diacetyllycorine (51)	“	“	“
Lycorine (55)	*Sternbergia lutea*	“	[81]
Methyl 2,4,6-trihydroxybenzoate (59)	*Cassia alata* L.	Not toxic	[84]
Aloe-emodin (60)	“	“	“
Kaempferol (61)		*M. oryzae* and *Phytophthora* sp.	
(-)-Epiafzelechin (62)	“	“	“
Rhein (63)	“	*Acidovorax avenae* subsp. *cattlvaePhytophthora* sp.	“
Kaempferol-3-*O*-glycoside (64)	“	*M. oryzae* and *Phytophthora* sp.	“
Kaempferol-3-*O*-gentiobiside (65)	“	“	“
Aloe-emodin-8-*O*-β-D-glucoside (66)	“	“	“

“ means the same content.

## 5. Conclusions

Biotic (pathogens) and abiotic stresses (climate changes) are the leading causes of heavy yield loss, severely damaging agricultural plants and, thus, food production. Similar effects were also observed on forest heritage and, consequently, on wood industries and nurseries. To address these challenges, there is an urgent need to reduce dependence on chemical pesticides, which have been extensively used over the past five to six decades. Thus, eco-friendly methods for biological control plant diseases based on natural compounds are intensely investigated. This review explored the potential of metabolites derived from bacteria, fungi and plants as valuable tools for the biological control of phytopathogenic bacteria and, in some instances, fungi. It discussed the findings of structure–activity relationship studies, along with the modes of action and efficacy of specific specialized metabolites. In conclusion, metabolomics shows great promise in identifying and screening novel bioactive metabolites, which could accelerate the discovery of effective candidates for sustainable plant protection. By incorporating metabolomics into screening methods, we can hasten the development of more effective and environmentally friendly agricultural practices. However, key challenges remain for the practical application of these promising metabolites as natural bacteriocides. These challenges include scaling up production, ensuring their efficacy in formulations and developing protocols for their field application.

## Data Availability

The data source was obtained from the SciFinder research motor.

## References

[1] Strobel G.A. (1982). Phytotoxins. Annu. Rev. Biochem..

[2] Ballio A., Graniti A. (1991). Phytotoxins and their involvement in plant diseases. Experientia.

[3] Bender C.L. (1998). Bacterial phytotoxins. Methods Microbiol..

[4] Durbin R. (2012). Toxins in Plant Disease.

[5] Duke S.O., Dayan F.E. (2011). Modes of action of microbially-produced phytotoxins. Toxins.

[6] Dudler R. (2012). The role of bacterial phytotoxins in inhibiting the eukaryotic proteasome. Trends Microbiol..

[7] Thakur A., Sharma V., Thakur A. (2018). Phytotoxins—A mini review. J. Pharmacogn. Phytochem..

[8] Chen H., Singh H., Bhardwaj N., Bhardwaj S.K., Khatri M., Kim K.H., Peng W. (2022). An exploration on the toxicity mechanisms of phytotoxins and their potential utilities. Crit. Rev. Environ. Sci. Technol..

[9] Tringali C. (2000). Bioactive Compounds from Natural Sources: Isolation, Characterization and Biological Properties.

[10] García-Pajón C.M., Collado I.G. (2003). Secondary metabolites isolated from *Colletotrichum* species. Nat. Prod. Rep..

[11] Cimmino A., Masi M., Evidente M., Superchi S., Evidente A. (2015). Fungal phytotoxins with potential herbicidal activity: Chemical and biological characterization. Nat. Prod. Rep..

[12] Evidente A., Cimmino A., Masi M. (2019). Phytotoxins produced by pathogenic fungi of agrarian plants. Phytochem. Rev..

[13] Dayan F.E., Duke S.O. (2014). Natural compounds as next-generation herbicides. Plant Physiol..

[14] Evidente A. (2023). Specialized metabolites produced by phytotopatogen fungi to control weeds and parasite plants. Microorganisms.

[15] Shettally R., Prasad R., Walia D.S. (1997). Effect of chemical pesticides on the environment. Indian J. Environ..

[16] Srivastav A.L., Prasad M.N.V. (2020). Chemical fertilizers and pesticides: Role in groundwater contamination. Agrochemicals Detection, Treatment and Remediation.

[17] Oerke E.C. (2006). Crop losses to pests. J. Agric. Sci..

[18] Seiber J.N., Coats J., Duke S.O., Gross A.D. (2014). Biopesticides: State of the art and future opportunities. J. Agric. Food Chem..

[19] Khursheed A., Rather M.A., Jain V., Rasool S., Nazir R., Malik N.A., Majid S.A. (2022). Plant based natural products as potential ecofriendly and safer biopesticides: A comprehensive overview of their advantages over conventional pesticides, limitations and regulatory aspects. Microb. Pathog..

[20] Macías-Rubalcava M.L., Sánchez-Fernández R.E. (2017). Secondary metabolites of endophytic *Xylaria* species with potential applications in medicine and agriculture. World J. Microbiol. Biotechnol..

[21] Tumbarski Y., Lante A., Krastanov A. (2018). Immobilization of bacteriocins from lactic acid bacteria and possibilities for application in food biopreservation. Open Biotechnol. J..

[22] Smits T.H., Duffy B., Blom J., Ishimaru C.A., Stockwell V.O. (2019). Pantocin A, a peptide-derived antibiotic involved in biological control by plant-associated *Pantoea* species. Arch. Microbiol..

[23] Vicente T.F., Félix C., Félix R., Valentão P., Lemos M.F. (2022). Seaweed as a natural source against phytopathogenic bacteria. Mar. Drugs.

[24] Akmukhanova N.R., Leong Y.K., Seiilbek S.N., Konysbay A., Zayadan B.K., Sadvakasova A.K., Sarsekeyeva F.K., Bauenova M.O., Bolatkhan K., Alharby H.F. (2023). Eco-friendly biopesticides derived from CO_2_-Fixing cyanobacteria. Environ. Res..

[25] Surico G., Comai L., Kosuge T. (1984). Pathogenicity of strains of *Pseudomonas syringae* pv. *savastanoi* and their indoleacetic acid deficient mutants on olive and oleander. Phytopathology.

[26] Surico G., Evidente A., Iacobellis N.S., Randazzo G. On the presence and level of different cytokinins in culture filtrate of *Pseudomonas syringae* pv. *savastanoi*. Proceedings of the Plant Pathogenic Bacteria: Proceedings of the Sixth International Conference on Plant Pathogenic Bacteria.

[27] Sisto A., Cipriani M.G., Morea M. (2004). Knot formation caused by *Pseudomonas syringae* subsp. savastanoi on olive plants is hrp-dependent. Phytopathology.

[28] Lavermicocca P., Lonigro S.L., Evidente A., Andolfi A. (1999). Bacteriocin production by *Pseudomonas syringae* pv. *ciccaronei* NCPPB2355. Isolation and partial characterization of the antimicrobial compound. J. Appl. Microbiol..

[29] Lavermicocca P., Lonigro S.L., Valerio F., Evidente A., Visconti A. (2002). Reduction of olive knot disease by a bacteriocin from *Pseudomonas syringae* pv. *ciccaronei*. Appl. Environ. Microbiol..

[30] Evidente A. (2022). Bioactive lipodepsipeptides produced by bacteria and fungi. Int. J. Mol. Sci..

[31] Bassarello C., Lazzaroni S., Bifulco G., Lo Cantore P., Iacobellis N.S., Riccio R., Gomez-Paloma L., Evidente A. (2004). Tolaasins A−E, five new lipodepsipeptides produced by *Pseudomonas tolaasii*. J. Nat. Prod..

[32] Wong W.C., Preece T.F. (1979). Identification of *Pseudomonas tolaasii*: The white line in agar and mushroom tissue block rapid pitting tests. J. Appl. Bacteriol..

[33] Lo Cantore P., Lazzaroni S., Coraiola M., Serra M.D., Cafarchia C., Evidente A., Iacobellis N.S. (2006). Biological characterization of white line-inducing principle (WLIP) produced by *Pseudomonas reactans* NCPPB1311. Mol. Plant Microbe Interact..

[34] Mieczkowski A., Speina E., Trzybiński D., Winiewska-Szajewska M., Wińska P., Borsuk E.M., Podsiadła-Białoskórska M., Przygodzki T., Drabikowski K., Stanczyk L. (2021). Diketopiperazine-based, flexible tadalafil analogues: Synthesis, crystal structures and biological activity profile. Molecules.

[35] Stierle A.C., Cardellina J.H., Strobel G.A. (1988). Maculosin, a hostspecific phytotoxin for spotted knapweed from *Alternaria alternata*. Proc. Natl. Acad. Sci. USA.

[36] Puopolo G., Cimmino A., Palmieri M.C., Giovannini O., Evidente A., Pertot I. (2014). *Lysobacter capsici* AZ78 produces cyclo (L-ProL-Tyr), a 2, 5-diketopiperazine with toxic activity against sporangia of *Phytophthora infestans* and *Plasmopara viticola*. J. Appl. Microbiol..

[37] Yamaguchi T. (1994). Horticulture in Japan.

[38] Young J.M., Fletcher M.J. (1994). *Pseudomonas syringae* pv. panici (Elliott 1923) Young, Dye & Wilkie 1978 is a doubtful name. Australas. Plant Pathol..

[39] Liu H., Qiu H., Zhao W., Cui Z., Ibrahim M., Jin G., Li B., Zhu B., Xie G.L. (2012). Genome sequence of the plant pathogen *Pseudomonas syringae* pv. panici LMG 2367. J. Bacteriol..

[40] Mapuranga N. (1998). A new race of *Pseudomonas syringae* pv. tabaci on tobacco in Zimbabwe. Plant Dis..

[41] Gutiérrez-Barranquero J.A., Cazorla F.M., de Vicente A. (2019). *Pseudomonas syringae* pv. syringae associated with mango trees, a particular pathogen within the “hodgepodge” of the Pseudomonas syringae complex. Front. Plant Sci..

[42] Young J.M. (1992). *Pseudomonas syringae* pv. *japonica* (Mukoo 1955) Dye et al. 1980 is a junior synonym of *Ps. syringae* pv. syringae van Hall 1902. Lett. Appl. Microbiol..

[43] Castaldi S., Cimmino A., Masi M., Evidente A. (2022). Bacterial lipodepsipeptides and some of their derivatives and cyclic dipeptides as potential agents for biocontrol of pathogenic bacteria and fungi of agrarian plants. J. Agric. Food Chem..

[44] Böszörményi E., Érsek T., Fodor A., Fodor A.M., Földes L.S., Hevesi M., Hogan J.S., Katona Z., Klein M.G., Kormany A. (2009). Isolation and activity of *Xenorhabdus* antimicrobial compounds against the plant pathogens *Erwinia amylovora* and *Phytophthora nicotianae*. J. Appl. Microbiol..

[45] Trippe K., McPhail K., Armstrong D., Azevedo M., Banowetz G. (2013). *Pseudomonas fluorescens* SBW25 produces furanomycin, a non-proteinogenic amino acid with selective antimicrobial properties. BMC Microbiol..

[46] McPhail K.L., Armstrong D.J., Azevedo M.D., Banowetz G.M., Mills D. (2010). I: 4-Formylaminooxyvinylglycine, an herbicidal germination-arrest factor from *Pseudomonas rhizosphere* bacteria. J. Nat. Prod..

[47] Kimbrel J.A., Givan S.A., Halgren A.B., Creason A.L., Mills D.I., Banowetz G.M., Armstrong D.J., Chang J. (2010). H: An improved, high-quality draft genome sequence of the Germination-Arrest Factor-producing *Pseudomonas fluorescens* WH6. BMC Genom..

[48] Mitchell R.E., Frey E.J., Benn M.H. (1986). Rhizobitoxine and L-threo-hydroxythreonine production by the plant pathogen *Pseudomonas andropogonis*. Phytochemistry.

[49] Sahm U., Knobloch G., Wagner F. (1973). Isolation and characterization of the methionine antagonist L-2-amino-4-methoxy-*trans*-3-butenoic acid from *Pseudomonas aeruginosa* grown on *n*-paraffin. J Antibiot..

[50] Braun S.D., Völksch B., Nüske J., Spiteller D. (2008). 3-Methylarginine from *Pseudomonas syringae* pv. syringae 22d/93 suppresses the bacterial blight caused by its close relative *Pseudomonas syringae* pv. glycinea. Chem. Bio Chem..

[51] Katagiri K., Tori K., Kimura Y., Yoshida T., Nagasaki T., Minato H. (1967). A new antibiotic. Furanomycin, an isoleucine antagonist. J. Med. Chem..

[52] Xie Y., Peng Q., Ji Y., Xie A., Yang L., Mu S., Li Z., He T., Xiao Y., Zhao J. (2021). Isolation and identification of antibacterial bioactive compounds from *Bacillus megaterium* L2. Front. Microbiol..

[53] Liu C., Bai L., Cao P., Li S., Huang S.X., Wa J., Li L., Zhang J., Zhao J., Song J. (2022). Novel plant growth regulator guvermectin from plant growthpromoting rhizobacteria boosts biomass and grain yield in rice. J. Agric. Food Chem..

[54] Zhang M., Li L., Li C., Ma A., Li J., Yang C., Chen X., Cao P., Li S., Zhang Y. (2004). Natural product guvermectin inhibits guanosine 5′-monophosphate synthetase and confers broad-spectrum antibacterial activity. Int. J. Biol. Macromol..

[55] Aghdam N.M.N., Baghaee-Ravari S., Shiri A. (2024). Weeds associated bacterial endophyte producing pantocin against *Pectobacterium carotovorum* subsp. *carotovorum*. J. Agric. Sci. Tech..

[56] Evidente A., Berestetskiy A., Cimmino A., Tuzi A., Superchi S., Melck D., Andolfi A. (2009). Papyracillic acid, a phytotoxic 1,6-dioxaspiro [4,4] nonene produced by *Ascochyta agropyrina* var. nana, a potential mycoherbicide for *Elytrigia repens* biocontrol. J. Agric. Food Chem..

[57] Hansske F., Sterner O., Satadler M., Anke H., Dorge L., Shan R. (1999). Papyracillic Acid, Method for Preparation and Its Use as Synthon for Bioactive Substances. U.S. Patent.

[58] Shan R., Heidren A., Stadler M., Sterner O. (1996). Papyracillic acid, a new penicillic acid analogue from the ascomycete *Lachnum papyraceum*. Tetrahedron.

[59] Evidente A. (2024). The incredible story of ophiobolin A and sphaeropsidin A: Two fungal terpenes from wilt-inducing phytotoxins to promising anticancer compounds. Nat. Prod. Rep..

[60] Ingels A., Scott R., Hooper A.R., van der Westhuyzen A.E., Wagh S.B., de Meester J., Maddau L., Marko D., Aichinger G., Berger W. (2024). New hemisynthetic derivatives of sphaeropsidin phytotoxins triggering severe endoplasmic reticulum swelling in cancer cells. Sci. Rep..

[61] Akatsuka T., Kodama O., Kato H., Kono Y., Takeuchi S. (1983). Short Communication 3-Hydroxy-7-oxo-sandaracopimaradiene (oryzalexin A), a new phytoalexin Isolated from rice blast leaves. Agric. Biol. Chem..

[62] Kono Y., Takeuchi S., Kodama O., Akatsuka T. (1984). Absolute configuration of oryzalexin A and structures of its related phytoalexins isolated from rice blast leaves infected with *Pyricularia oryzae*. Agric. Biol. Chem..

[63] Akatsuka T., Kodama O., Sekido H., Kono Y., Takeuchi S. (1985). Novel phytoalexins (oryzalexins A, B and C) isolated from rice blast leaves infected with *Pyricularia oryzae*. Part I: Isolation, characterization and biological activities of oryzalexins. Agric. Biol. Chem..

[64] Cartwright D., Langcake P., Pryce R.J., Leworthy D.P., Ride J.P. (1977). Chemical activation of host defence mechanisms as a basis for crop protection. Nature.

[65] Evidente A., Venturi V., Masi M., Degrassi G., Cimmino A., Maddau L., Andolfi A. (2011). In vitro antibacterial activity of sphaeropsidins and chemical derivatives toward *Xanthomonas oryzae* pv. oryzae, the causal agent of rice bacterial blight. J. Nat. Prod..

[66] Li S., Shao M.W., Lu Y.H., Kong L.C., Jiang D.H., Zhang Y.L. (2014). Phytotoxic and antibacterial metabolites from *Fusarium proliferatum* ZS07 isolated from the gut of long-horned grasshoppers. J. Agric. Food Chem..

[67] Ngo M.T., Van Nguyen M., Han J.W., Kim B., Kim Y.K., Park M.S., Kim H., Choi G.J. (2021). Biocontrol potential of *Aspergillus* species producing antimicrobial metabolites. Front. Microbiol..

[68] Cimmino A., Pescitelli G., Berestetskiy A., Dalinova A., Krivorotov D., Tuzi A., Evidente A. (2016). Biological evaluation and determination of the absolute configuration of chloromonilicin, a strong antimicrobial metabolite isolated from *Alternaria sonchi*. J. Antibiot..

[69] Padhi S., Masi M., Panda S.K., Luyten W., Cimmino A., Tayung K., Evidente A. (2020). Antimicrobial secondary metabolites of an endolichenic *Aspergillus niger* isolated from lichen thallus of *Parmotrema ravum*. Nat. Prod. Res..

[70] van Der Wolf J.M., Nijhuis E.H., Kowalewska M.J., Saddler G.S., Parkinson N., Elphinstone J.G., Pritchard L., Toth I.K., Lojkowska E., Potrykus M. (2014). *Dickeya solani* sp. nov., a pectinolytic plant-pathogenic bacterium isolated from potato (*Solanum tuberosum*). Int. J. Syst. Evol. Microbiol..

[71] Takikawa Y., Takahashi F. (2014). Bacterial leaf spot and blight of crucifer plants (Brassicaceae) caused by *Pseudomonas syringae* pv. *maculicola* and *P. cannabina* pv. *alisalensis*. J. Gen. Plant. Pathol..

[72] Vieira G., Khalil Z.G., Capon R.J., Sette L.D., Ferreira H., Sass D.C. (2022). Isolation and agricultural potential of penicillic acid against citrus canker. J. Appl. Microbiol..

[73] Wei L., Zhang Q., Xie A., Xiao Y., Guo K., Mu S., Xie Y., Li Z., He T. (2022). Isolation of bioactive compounds, antibacterial activity, and action mechanism of spore powder from *Aspergillus niger* xj. Front. Microbiol..

[74] Avrova A.O., Hyman L.J., Toth R.L., Toth I.K. (2002). Application of amplified fragment length polymorphism fingerprinting for taxonomy and identification of the soft rot bacteria *Erwinia carotovora* and *Erwinia chrysanthemi*. Appl. Environ. Microbiol..

[75] Fuller S.L., Savory E.A., Weisberg A.J., Buser J.Z., Gordon M.I., Putnam M.L., Ghang J.H. (2017). Isothermal amplification and lateral-flow assay for detecting crown-gall-causing *Agrobacterium* spp.. Phytopathology.

[76] Mansfield J., Genin S., Magori S., Citovsky V., Sriariyanum M., Ronald P., Dow M., Verdier V., Beer S.V., Machados M.A. (2012). Top 10 plant pathogenic bacteria in molecular plant pathology. Mol. Plant Pathol..

[77] Schrader K.K., Dayan F.E., Allen S.N., de Regt M.Q., Tucker C.S., Paul R.N. (2000). 9,10-Anthraquinone reduces the photosynthetic efficiency of *Oscillatoria perornata* and modifies cellular inclusions. Int. J. Plant Sci..

[78] Klesius P.H., Evans J., Shoemaker C. (2006). Advancements in fish vaccine development. Aquac Int..

[79] Boyd C.E., Tucker C.S. (1998). Pond Aquaculture Water Quality Management.

[80] Schrader K.K., Andolfi A., Cantrell C.L., Cimmino A., Duke S.O., Osbrink W., Wedge E.W., Evidente A. (2010). A survey of phytotoxic microbial and plant metabolites as potential natural products for pest management. Chem. Biodivers..

[81] Kornienko A., Evidente A. (2008). Chemistry, biology, and medicinal potential of narciclasine and its congeners. Chem. Rev..

[82] Schrader K.K., Avolio F., Andolfi A., Cimmino A.ì., Evidente A. (2013). Ungeremine and its hemisynthesized analogs as bactericides against *Flavobacterium columnare*. J. Agric. Food Chem..

[83] Simirgiotis M.J., Burton D., Parra F., López J., Muñoz P., Escobar H., Parra C. (2020). Antioxidant and antibacterial capacities of *Origanum vulgare* L. essential oil from the arid Andean Region of Chile and its chemical characterization by GC-MS. Metabolites.

[84] Pham D.Q., Pham H.T., Han J.W., Nguyen T.H., Nguyen H.T., Nguyen T.D., Nguyen T.T.T., Cuong T.H., Pham H.M., Vu H.D. (2021). Extracts and metabolites derived from the leaves of *Cassia alata* L. exhibit in vitro and in vivo antimicrobial activities against fungal and bacterial plant pathogens. Ind. Crops. Prod..

[85] Dongmo A.N., Nguefack J., Dongmo J.B.L., Fouelefack F.R., Azah R.U., Nkengfack E.A., Stefani E. (2021). Chemical characterization of an aqueous extract and the essential oil of *Tithonia diversifolia* and their biocontrol activity against seed-borne pathogens of rice. J. Plant Dis. Prot..

